# Six-Month Effectiveness of Advanced *vs.* Standard Hybrid Closed-Loop System in Children and Adolescents With Type 1 Diabetes Mellitus

**DOI:** 10.3389/fendo.2021.766314

**Published:** 2021-11-09

**Authors:** Gianluca Tornese, Francesca Buzzurro, Claudia Carletti, Elena Faleschini, Egidio Barbi

**Affiliations:** ^1^ Institute for Maternal and Child Health IRCCS “Burlo Garofolo”, Trieste, Italy; ^2^ University of Trieste, Trieste, Italy

**Keywords:** hybrid closed loop, glycemic control, advanced, children, adolescents, HbA1c

## Abstract

**Introduction:**

The purpose of this study was to assess the effectiveness of advanced- (a-HCL) *vs.* standard-hybrid closed-loop (s-HCL) systems use up to 6 months of treatment in a real-world setting of children and adolescents with T1DM.

**Methods:**

We retrospectively evaluated all T1DM pediatric users of MiniMed™ 670G system (s-HCL) and 780G system (a-HCL). HbA1c and BMI were collected at baseline and three and six months after HCL start. Data on glycemic control were extracted from reports generated with CareLink™ Personal Software in Manual Mode, at HCL start, after one, three, and six months after HCL beginning.

**Results:**

The study included 44 individuals with a median age of 13.3 years (range 2- 21 years), 20 on s-HCL, and 24 on a-HCL. a-HCL users had a significantly lower HbA1c compared to s-HCL after six months of HCL use (7.1 *vs.* 7.7%). Significant differences in HbA1c between a-HCL and s-HCL users were found in children aged 7-14 years (7.1 *vs.* 7.7% after six months) and in those with a worse (HbA1c >8%) glycemic control at the beginning (7.1 *vs.* 8.1% after six months). No significant changes in HbA1c were found in a-HCL users that previously used a s-HCL system. Nevertheless, only the use of a-HCL significantly predicted a lower HbA1c after six months. All sensor-specific measures of glycemic control improved from Manual to Auto mode, in both s-HCL and a-HCL, without increasing time spent in hypoglycemia. However, the percentage of individuals with TIR>70% increased significantly in a-HCL users, who attained this target earlier and more stably: younger age, a higher rate of auto-correction, and a lower amount of CHO inserted predicted a TIR>70%. BMI SDS did not significantly change throughout the study period.

**Conclusion:**

This real-world study suggests that effectiveness might be greater in a-HCL than in s-HCL, with significant changes in HbA1c, and reaching earlier and more stably the target of TIR >70%, without increasing hypoglycemia or BMI. At the same time, previous users of s-HCL systems did not show any further improvement with a-HCL. Children under the age of 14 years of age, not represented in previous studies, seem to benefit the most from a-HCL pumps as well as individuals with the worst glycemic control.

## Introduction

Individuals with type 1 diabetes mellitus (T1DM) face daily hardships due to the rigorous management of insulin replacement therapy, necessary to control blood glucose levels. This task is especially challenging for children and adolescents (1–4). Over the past twenty years, efforts have been made to improve the quality of life of these patients and their families by introducing new technologies. These endeavors have been mainly focused on developing devices capable of replacing the normal function of the pancreas, which may be collectively referred to as artificial pancreas technologies. Three fundamental components constitute hybrid closed-loop (HCL) systems: a sensor for continuous glucose monitoring (CGM), pumps necessary for continuous subcutaneous insulin infusion (CSII), and an algorithm for automated insulin delivery, increasing or suspending basal insulin infusion based on glucose values wirelessly transmitted by the sensor (5, 6). These systems are called “hybrid” since pre-prandial boluses are not fully automatized: boluses must be delivered by the users before the meal, calculating insulin units on pre-prandial blood glucose level and the grams of carbohydrates to be consumed.

The first commercialized standard hybrid closed-loop (s-HCL) system (Medtronic MiniMed™ 670G) infuses micro boluses of insulin to reach a glucose target of 120 mg/dL. The pivotal trial showed a 5.5% increase in time in range (TIR, between 70 and 180 mg/dL) (7, 8), and real-life data showed improved outcomes and the superiority of 670G compared to any other treatment modality (multi-daily injections [MDI], CSII or sensor-augmented pump [SAP]) (9–13). One real-life study on 92 individuals between 2-25 years of age found a decrease in HbA1c of -0.3% after six months of 670G use (14), while another study on 111 children and adolescents aged 3 to 16 years found an improvement in sensor-specific measures of glycemic control (but not HbA1c) that lasted throughout the first year of treatment (15).

In October 2020, an advanced hybrid closed-loop (a-HCL) system (Medtronic MiniMed™ 780G) was available in many European countries. The 780G includes an algorithm with individualized basal target set points (100, 110, and 120 mg/dL) and autocorrection insulin boluses to a fixed glucose target of 120 mg/dL (16–18). The pivotal trial showed a TIR of 74.5% after three months of using the 780G system in 157 adolescents and adults with T1D, with improved outcomes reached using the most aggressive algorithm (glucose target of 100 mg/dL) (19). A randomized crossover trial has shown, over 12 + 12 weeks, a reduction in time in hyperglycemia, without increasing hypoglycemia, when compared to the 670G s-HCL system in 113 adolescents and young adults, aged between 14 and 29 years (20). A recent real-life study on 52 T1DM adolescents and adults aged between 15 and 65 years, using a-HCL, showed an immediate improvement in glycemic control with a 12.3% increase in TIR in the first month in well-controlled and experienced SAP with predictive low glucose suspension (PLGS) users (21).

Although data from clinical trials are encouraging, real-world studies on HCL use in children and adolescents are needed to assess the effectiveness of a-HCL *vs.* s-HCL and should include changes in longer-term glycemic outcomes, such as HbA1c.

The purpose of this study was to examine real-world data on both s-HCL, and a-HCL systems use up to 6 months of treatment in a clinical population of children and adolescents with T1DM.

## Materials and Methods

We retrospectively evaluated all individuals with T1DM followed at the Diabetes Pediatric Unit of the Institute for Maternal and Child Health “Burlo Garofolo” (an academic tertiary hospital and research institute that serves as a pediatric referral center for the province of Trieste, Italy) who started using an HCL system (s-HCL Medtronic MiniMed™ 670G from February 1^st^, 2019 until September 29^th^, 2020 or a-HCL Medtronic MiniMed™ 780G from October 26^th^, 2020 until April 1^st^, 2021) and had at least six months of follow-up. HCL system was also used in children <7 years of age (but with a minimum daily dose ≥of 8 units) if the clinician considered this treatment to be beneficial and the family agreed on off-label use of the system.

In our current practice (22, 23), individuals naïve to CSII are required to wear a standalone CGM for two weeks while on MDI. The training [as previously described (23)] included two weeks using the HCL system in Manual Mode (with a single basal rate over 24 hours, dividing the units of long-acting basal insulin by 24), followed by Auto Mode. All individuals with T1DM in our Institute are instructed by a dietician to carbohydrates (CHO) counting since T1DM onset.

We extracted data on glycemic control from reports generated with CareLink™ Personal Software with observation time frames of 2 weeks: Manual Mode (MANUAL), beginning of Auto Mode (AUTO START), after one month (AUTO 1 MONTH), after three months (AUTO 3 MONTHS) and after six months of Auto mode (AUTO 6 MONTHS).

The “G2 clinico” platform (management system specialist activities) was employed to access patients’ clinical data. Information retrieved included sex, age at T1DM onset, previous insulin treatment and HbA1c, weight and BMI standard deviation score (SDS) for the last visit before HCL training (BASELINE), the closest visit at three months of HCL use (3 MONTHS), and that at six months (6 MONTHS). HbA1c was measured with point-of-care testing (DCA2000+, Siemens). The BMI SDS was determined employing Growth Calculator 3 Software using Italian reference charts (24).

Ethical Committee approval was not requested since General Authorization to Process Personal Data for Scientific Research Purposes (Authorization no. 9/2014) declared that retrospective archive studies that use ID codes, preventing the data from being traced back directly to the data subject, do not need ethics approval (25). Parents signed informed consent at the first visit, in which they agreed that “clinical data may be used for clinical research purposes, epidemiology, the study of pathologies and training, to improve knowledge, care, and prevention.” In addition, all parents were requested to give specific informed consent for the collection of the data.

All statistical analyses were conducted with JMP™ (version 16.1.0, SAS Institute Inc., Cary, NC, United States). Data are presented as median and interquartile ranges (IQRs). Wilcoxon signed-rank test was performed to check whether the differences between paired data were statistically significant. Single-linear regression and multivariate logistics regressions were carried out to study associations between a dichotomous outcome and one or more independent variables. A p-value <0.05 was considered statistically significant. Fixing alpha=0.05 and beta=0.20, supposing a paired mean difference between pre-post HbA1 equal to 0.5% (SD=1, effect size=0.50), a sample size of 27 subjects was needed.

## Results

This retrospective study included 44 individuals (27 females) with T1DM [median age at diagnosis 7.7 years (IQR 4.9;11.6)]: 20 users of MiniMed™ 670G (s-HCL), and 24 users of MiniMed™ 780G (a-HCL). Baseline characteristics are reported in **Table 1**.

**Table 1:** Baseline characteristics for the entire study cohort and divided between a-HCL and s-HCL users.

	All (n = 44)	s-HCL (n = 20)	a-HCL (n = 24)	p _s-HCL *vs*. a-HCL_
**Female sex**	27 (61%)	12 (60%)	15 (63%)	*1.00*
**Age at HCL start** (years)	13.7 (9.2; 15.3)	13.1 (9.6; 15.1)	14.1 (9.2; 15.9)	*0.64*
*<7 years (n, %)*	5 (12%)	1 (5%)	4 (17%)	*0.25*
*7-14 years (n, %)*	17 (38%)	10 (50%)	7 (29%)	
*>14 years (n, %)*	22 (50%)	9 (45%)	13 (54%)	
**BMI** (SDS)	0.42 (-0.35; 1.48)	0.57 (-0.40; 1.48)	0.40 (-0.23; 1.60)	*0.98*
**T1DM duration** (years)	4.1 (1.5; 7.5)	5.3 (2.1; 7.5)	3.1 (1.1; 7.6)	*0.42*
**Naïve to CSII**	20 (45%)	10 (50%)	10 (41%)	*0.54*
**Baseline HbA1c**	7.9 (7.2; 8.3)	8.0 (6.9; 8.3)	7.8 (7.2; 8.5)	*0.94*
*<7%*	8 (18%)	5 (25%)	3 (12%)	*0.50*
*7-8%*	16 (36%)	6 (30%)	10 (42%)	
*>8%*	20 (46%)	9 (45%)	11 (46%)	

a-HCL, advanced Hybrid Closed Loop; BMI, Body Mass Index; CSII, continuous subcutaneous insulin infusion; HCL, Hybrid Closed Loop; s-HCL, standard Hybrid Closed Loop; SDS, standard deviation score; T1DM, Type 1 Diabetes Mellitus. Data are reported as number and percentage or median and interquartile range.

Training for the HCL system started at a median age of 13.3 years (IQR 8.9;15.2; range 2-21 years): 5 individuals were <7 years of age [median age of 5.5 years (IQR 3.6;6.2)], 17 between 7 and 14 years [median age of 10.0 years (IQR 9.1;13.1)], and 22 were >14 years [median age of 15.3 years (IQR 14.4;17.3)].

Median HbA1c at the last visit before HCL training (BASELINE) was 7.9%. It was significantly higher in individuals naïve to insulin pumps [8.2% (IQR 7.5;8.6)] than those who were already using another pump [7.4% (IQR 6.9;8.3)]. No significant differences at baseline were found between s-HCL and a-HCL users (**Table 1**). Age and sex were not associated with any of the variables.

The visit at three months of HCL use with HbA1c evaluation (3 MONTHS) was at a median of 91 days (IQR 71;111) from the beginning of HCL for s-HCL users and 89 days (IQR 61;125) for a-HCL users (p=0.94). The visit at six months of HCL use with HbA1c evaluation (6 MONTHS) was at a median of 194 days (IQR 171;225) from the beginning of HCL for s-HCL users and 193 days (IQR 175;230) for a-HCL users (p=0.95).

No patients discontinued system use during the study period.

### Changes in HbA1c

**Figure 1:**
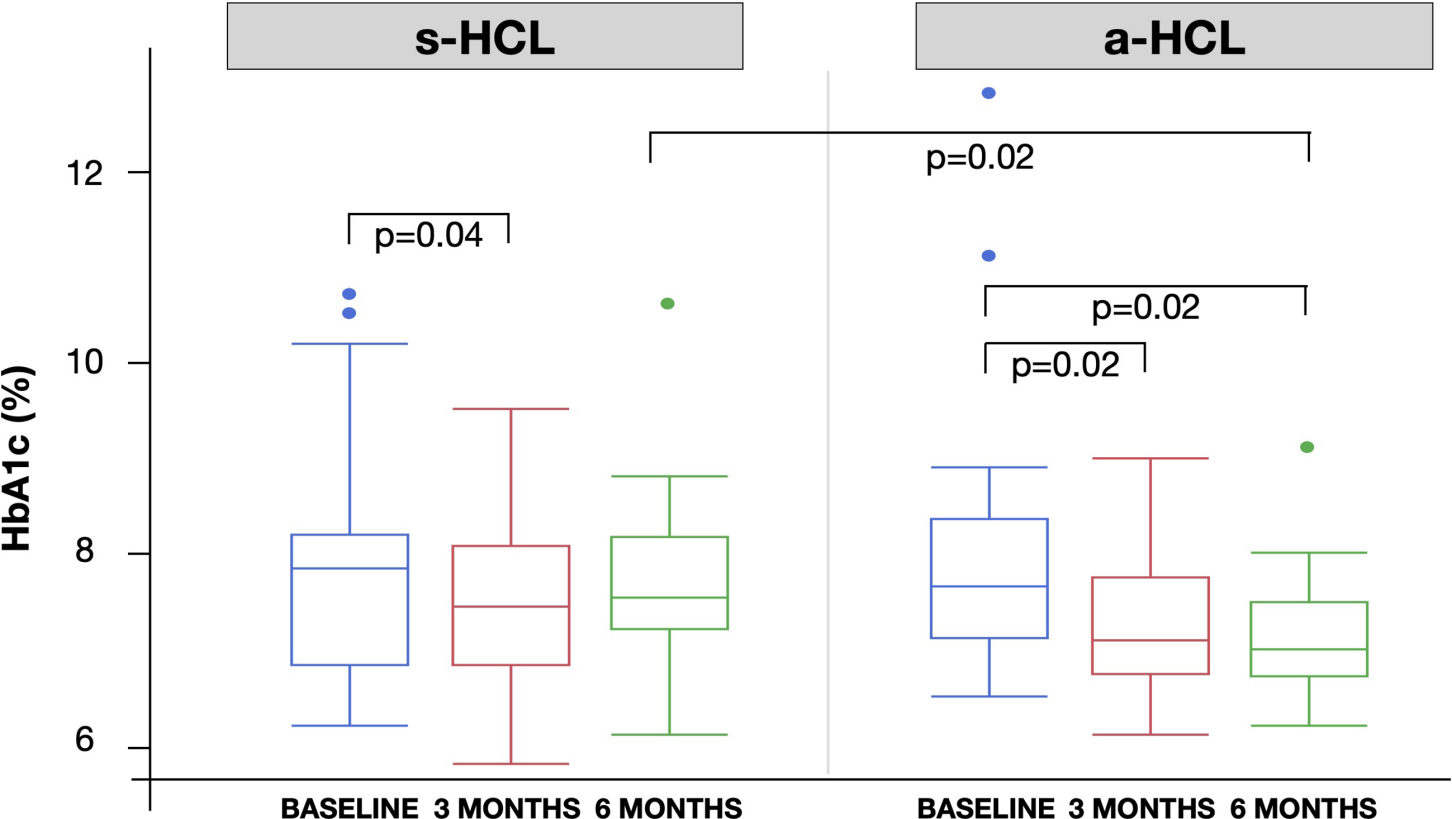
Box plot with the distribution of HbA1c across the 3 study time: BASELINE (last visit before use of HCL), 3 MONTHS and 6 MONTHS after first visit after the beginning of HCL use.

HbA1c was significantly lower at 3 MONTHS compared to BASELINE for both s-HCL users (from 8.0% to 7.6%, p=0.04) and a-HCL users (from 7.8% to 7.2%, p=0.02). HbA1c at 6 MONTHS in a-HCL users was significantly lower compared to BASELINE (7.1 vs. 7.8%, p=0.02) and compared to s-HCL users (7.1 *vs.* 7.7%, p=0.02). (**Figure 1** and **Table 2**).

**Table 2:** HbA1c at the visit 3 months (3 MONTHS) and 6 months (6 MONTHS) after HCL start, compared to BASELINE (last visit before use of HCL) and change in HbA1c (ΔHbA1c) between time points for s-HCL and a-HCL users and according to age classes, previous insulin treatment and glycemic control before HCL start.

		HbA1c BASELINE	HbA1c 3 MONTHS	ΔHbA1c _3 MONTHS-BASELINE_	p _3 MONTHS-BASELINE_	HbA1c 6 MONTHS	ΔHbA1c _6 MONTHS-BASELINE_	p _6 MONTHS-BASELINE_	p _6 MONTHS-3 MONTHS_
***Type of HCL system***									
*s-HCL (n=20)*		8.0 (6.9;8.3)	7.6 (6.9;8.3)	-0.2 (-1.0;0.3)	**0.04**	7.7 (7.3;8.3)^§^	-0.1 (-0.7;0.3)	0.23	0.24
*a-HCL (n=24)*		7.8 (7.2;8.5)	7.2 (6.9;7.9)	-0.4 (-1.0;-0.1)	**0.02**	7.1 (6.8;7.6)^§^	-0.5 (-1.4;-0.1)	**0.02**	0.51
***+ age classes***									
*s-HCL*	*<7 years (n=1)*	8.5	7.0	-1.5	–	7.6	-0.9	–	**-**
	*7-14 years (n=10)*	8.0 (7.2;8.2)	7.7 (7.1;8.3)	0.0 (-0.6;0.3)	0.42	7.7 (7.4;8.4)*	0.0 (-0.5;0.4)	0.74	0.59
	*>14 years (n=9)*	7.5 (6.8;9.3)	7.3 (6.8;8.2)	-0.4 (-1.6;0.3)	0.13	7.3 (6.7;8.2)	-0.1 (-0.8;0.3)	0.34	0.42
	**p**	0.52	0.54	0.29		0.51	0.35		
*a-HCL*	*<7 years (n=4)*	7.8 (7.0;8.4)	7.2 (7.0:7.6)	-0.4 (-1.2;0.2)	0.28	7.2 (7.0;7.8)	-0.3 (-1.3;0.3)	0.40	0.60
	*7-14 years (n=7)*	8.1 (7.3;9.0)	7.2 (6.4;7.4)	-0.7 (-1.4;-0.4)	0.14	7.1 (6.7;7.7)*	-0.6 (-1.9;-0.1)	0.14	0.92
	*>14 years (n=13)*	7.7 (7.2;8.3)	7.3 (6.7;8.0)	-0.3 (-0.9;0.2)	0.13	7.1 (6.7;7.6)	-0.7 (-1.3;0.3)	0.14	0.46
	**p**	0.59	0.67	0.34		0.84	0.68		
***+ previous insulin treatment***									
*s-HCL*	*MDI (n=10)*	8.3 (7.5;10.4)	7.6 (7.0;8.3)	-0.9 (-1.7;0.0)	**0.01**	7.8 (7.2;8.5)	-0.4 (-1.1;0.1)	0.13	0.22
	*CSII (n=10)*	7.4 (6.8;8.2)	7.5 (6.9;8.1)	0.2 (-0.3;0.4)	0.86	7.6 (7.2;7.9)	0.0 (-0.4;0.6)	0.65	0.79
	**p**	0.06	0.73	**0.02**		0.40	0.10		
*a-HCL*	*MDI (n=9)*	8.1 (7.5;8.6)	7.1 (6.4;7.6)	-0.9 (-1.4;-0.3)	0.05	6.9 (6.6;7.9)	-0.9 (-1.5;-0.3)	0.09	0.54
	*CSII (n=6)*	7.5 (7.2;9.0)	7.0 (6.7;7.7)	-0.6 (-1.4;-0.4)	**0.04**	7.1 (6.8;7.2)	-0.6 (-1.9;-0.2)	0.12	0.44
	*s-HCL (n=9)*	7.4 (7.0;8.3)	7.7 (7.2;8.3)	0.2 (-0.3;0.6)	0.05	7.3 (7.1;7.8)	-0.1 (-1.0;0.6)	0.09	0.54
	**p**	0.54	**0.04**	**<0.01**		0.25	0.20		
***+ glycemic control before HCL start***									
*s-HCL*	*HbA1c <7% (n=5)*	6.7 (6.5;6.8)	6.8 (6.3;7.2)	0.1 (-0.2;0.4)	0.59	7.3 (6.6;7.6)	0.6 (0.1;0.8)	0.05	**<0.01**
	*HbA1c 7-8% (n=6)*	7.5 (7.3;7.7)	7.7 (7.2;8.0)	0.4 (-0.6;0.6)	0.73	7.5 (6.7;8.1)	-0.4 (-0.8;0.7)	0.75	0.55
	*HbA1c >8% (n=9)*	8.3 (8.3;10.4)	8.0 (7.2;8.6)	-1.0 (-1.7;-0.1)	**0.01**	8.1 (7.6;8.5)§	-0.3 (-1.1;0.0)#	0.05	0.28
	**p**	**<0.01**	**0.01**	**0.02**		0.05	**0.03**		
*a-HCL*	*HbA1c <7% (n=3)*	6.7 (6.6;6.8)	7.1 (6.4;7.2)	0.4 (-0.3;0.5)	0.51	7.3 (7.3;7.8)	0.7 (0.5;1.1)	**0.04**	0.30
	*HbA1c 7-8% (n=10)*	7.4 (7.2;7.6)	7.0 (6.7;7.5)	-0.4 (-0.8;-0.0)	0.07	7.1 (6.6;7.4)	-0.3 (-0.6;0.1)	0.42	0.46
	*HbA1c >8% (n=11)*	8.5 (8.2;9.0)	7.7 (7.2;8.6)	-0.7 (-1.4;-0.4)	0.05	7.1 (6.8;7.9)§	-1.4 (-1.9;-0.7)#	**<0.01**	0.08
	**p**	**<0.01**	0.05	0.06		0.36	**<0.01**		

Bold values mean significant difference. Statistically significant differences between s-HCL vs. a-HCL in subgroups § HbA1c 6 MONTHS, p=0.02; *HbA1c 6 MONTHS in 7-14 years, p=0.03; §HbA1c 6 MONTHS in HbA1c>8% before HCL start, p<0.01; # ∆HbA1c 6 MONTHS-BASELINE in HbA1c>8% before HCL start, p=0.04.

a-HCL, advanced Hybrid Closed Loop; CSII, continuous subcutaneous insulin infusion; HCL, Hybrid Closed Loop; MDI, multi daily injections; s-HCL, standard Hybrid Closed Loop.

In children aged 7-14 years, HbA1c at 6 MONTHS was significantly lower in a-HCL than in s-HCL users (7.1% *vs.* 7.7%, p=0.03). A trend of HbA1c reduction from BASELINE to 3 MONTHS and 6 MONTHS was also noted in children <7 years and adolescents >14 years, without statistical significance (**Table 2**).

A significant reduction in HbA1c from BASELINE to 3 MONTHS was observed in those who were on MDI treatment before starting HCL (naïve to CSII) for s-HCL users (from 8.3% to 7.6%, p=0.01), and in previous users of other types of CSII (non-HCL) for a-HCL users (from 7.5% to 7.0%, p=0.04). No significant changes in HbA1c were found in a-HCL users that previously used a s-HCL system (**Table 2**).

When considering the glycemic control before HCL start (HbA1c at BASELINE), in individuals with the worst control (HbA1c >8%), s-HCL users had a significant reduction at 3 MONTHS (from 8.3 to 8.0%, p=0.01), while a-HCL users had a significant decrease at 6 MONTHS (from 8.5 to 7.1%, p<0.01). Both HbA1c at 6 MONTHS and the reduction in HbA1c from BASELINE to 6 MONTHS (ΔHbA1c _6 MONTHS-BASELINE_) were significantly improved in a-HCL users compared to s-HCL users (7.1 *vs.* 8.1%, p<0.01, and -1.4 *vs.* -0.3%, p=0.04, respectively). In individuals with the best control (HbA1c <7%) at BASELINE, HbA1c significantly increased at 6 MONTHS in s-HCL users (7.3 *vs.* 6.8% at 3 MONTHS, p<0.01) and in a-HCL users (7.3 *vs.* 6.7% at BASELINE, p=0.04). In a-HCL users, the difference in HbA1c among classes of pre-HCL glycemic control was not significant from 3 MONTHS onwards (**Table 2**).

A linear regression model, including age, sex, HbA1c at BASELINE, type of HCL, and naïveté to CSII, was used to examine associations with ΔHbA1c, and only the use of a-HCL (p=0.01) significantly predicted a lower HbA1c at 6 MONTHS.

### Changes in Sensor-Specific Measures of Glycemic Control

GMI (an approximation of HbA1c based on CGM readings), mean glucose sensor, TIR (sensor glycemic levels between 70 and 180 mg/dl), and TAR (both Level 1, 180-250 mg/dl, and Level 2, >250 mg/dl) improved significantly from MANUAL to all time points in both s-HCL and a-HCL users (except at 3 MONTHS for s-HCL). TBR (sensor glycemic levels <70 mg/dl) did not change significantly throughout the study period (except at AUTO START for s-HCL). No significant differences in coefficient of variation (CV) were observed throughout the study period (**Table 3**).

**Table 3:** Glycemic outcomes at the beginning (AUTO START), after 1 month (AUTO 1 MONTH), after 3 months (AUTO 3 MONTHS) and after 6 months (AUTO 6 months) of HCL beginning, compared to Manual mode (MANUAL) for s-HCL and a-HCL users.

	Type of HCL	MANUAL	AUTO START	p _AUTO START-MANUAL_	AUTO1 MONTH	p _AUTO 1 MONTH-MANUAL_	p _AUTO 1 MONTH-AUTO START_	AUTO 3 MONTHS	p _AUTO 3 MONTHS-MANUAL_	p _AUTO 3 MONTHS-AUTO START_	p _AUTO 3 MONTHS-AUTO 1 MONTH_	AUTO 6 MONTHS	p _AUTO 6 MONTHS-MANUAL_	p _AUTO 6 MONTHS-AUTO START_	p _AUTO 6 MONTHS-AUTO 1 MONTH_	p _AUTO 6 MONTHS-AUTO 3 MONTHS_
***Sensor-specific measures of glycemic control***																
GMI (%)	s-	7.4 (7.0;7.9)	7.1 (6.9;7.4)	***0.02***	7.0 (6.7;7.3)	***<0.01***	*0.28*	7.2 (6.9;7.3)	***<0.01***	***<0.01***	*0.19*	7.2 (6.9;7.2)	***<0.01***	***<0.01***	*0.31*	*0.43*
	a-	7.4 (7.0;8.0)	6.9 (6.7;7.1)	***<0.01***	6.9 (6.6;7.2)	***<0.01***	*0.33*	7.1 (6.7;7.4)	***<0.01***	***<0.01***	***0.03***	7.1 (6.7;7.2)	***<0.01***	***<0.01***	*0.50*	*0.19*
Mean sensor glucose (mg/dl)	s-	170 (153;191)	157 (148;170)§	***0.02***	155 (144;167)	***<0.01***	*0.11*	160 (149;166)	***0.01***	*0.89*	*0.14*	161 (152;164)	***0.04***	*0.86*	*0.08*	*0.67*
	a-	166 (152;188)	150 (140;158)§	***<0.01***	148 (139;162)	***<0.01***	*0.62*	154 (141;168)	***<0.01***	*0.08*	***0.02***	158 (141;162)	***<0.01***	*0.28*	*0.12*	*0.34*
Coefficient of variation (%)	s-	34.6 (31.1;36.4)	32.7 (30.4;35.3)	*0.28*	33.2 (30.5;37.0)	*0.60*	*0.70*	32.4 (30.0;35.8)	*0.19*	*0.67*	*0.41*	34 .5(29.7;37.6)	*0.25*	*0.81*	*0.90*	*0.48*
	a-	33.6 (30.3;38.0)	34.7 (29.8;36.1)	*0.43*	32.2 (29.9;36.8)	*0.31*	*0.74*	31.2 (29.6;36.7)	*0.07*	*0.37*	*0.43*	33.5 (30.7;38.0)	*0.97*	*0.61*	*0.45*	*0.14*
TAR (>180 mg/dl)	s-	39 (25;54)	31 (22;38)	***<0.01***	29 (20;37)	***<0.01***	*0.30*	31 (22;35)	***<0.01***	*0.86*	***0.40***	30 (27;36)	***0.01***	*0.95*	*0.29*	*0.87*
	a-	40 (29:54)	25 (18;34)	***<0.01***	24 (19;30)	***<0.01***	*0.18*	27 (19;35)	***<0.01***	*0.14*	***0.02***	28 (16;34)	***<0.01***	*0.90*	*0.19*	*0.26*
- Level 1 (180-250 mg/dl)	s-	28 (23;33)	23 (18;29)	***0.02***	24 (18;26)	***<0.01***	*0.34*	24 (19;28)	***0.01***	*0.65*	*0.71*	24 (20;27)	***<0.01***	*0.95*	*0.34*	*0.69*
	a-	29 (22;32)	22 (16;26)	***<0.01***	20 (18;25)	***<0.01***	*0.40*	22 (17;25)	***0.01***	*0.30*	*0.09*	22 (15;25)	***<0.01***	*0.38*	*0.96*	*0.18*
- Level 2 (>250 mg/dl)	s-	10 (5;19)	5 (3;10)	***0.02***	5 (2;10)	***<0.01***	*0.39*	7 (3;11)	***0.03***	*0.83*	*0.10*	7 (3;10)	***0.04***	*0.97*	*0.32*	*0.68*
	a-	9 (5;20)	4 (1;7)	***<0.01***	3 (2;7)	***<0.01***	*0.89*	5 (2;8)	***<0.01***	*0.08*	***0.01***	6 (1;9)	***<0.01***	*0.10*	*0.05*	*0.83*
TIR (70-180 mg/dl)	s-	60 (46;72)	67 (62;76)	***<0.01***	71 (63;78)	***<0.01***	*0.29*	67 (57;76)	*0.26*	*0.45*	*0.19*	69 (63;71)	***<0.01***	*0.92*	*0.31*	*0.43*
	a-	58 (46;70)	74 (64;82)	***<0.01***	74 (68;80)	***<0.01***	*0.29*	73 (63;79)	***<0.01***	*0.11*	***0.03***	72 (65;82)	***<0.01***	*0.93*	*0.50*	*0.19*
TBR (<70 mg/dl)	s-	1 (1;3)	1 (1;3)	*0.72*	1 (0;3)	***<0.01***	*0.49*	1 (0;2)	*0.70*	*0.89*	*0.56*	1 (0;3)	*0.51*	*0.64*	*0.55*	*0.88*
	a-	1 (0:3)	1 (0;3)	*0.86*	1 (0;4)	*0.83*	*0.67*	1 (0;3)	*0.91*	*1.00*	*0.64*	1 (0;2)	*0.23*	*0.27*	*0.17*	*0.27*
- Level 1 (54-70 mg/dl)	s-	1 (1;3)	1 (1;2)	*0.38*	1 (0;2)	*0.74*	*0.66*	1 (0;2)	*0.43*	*0.86*	*0.37*	1 (0;2)	*0.20*	*0.37*	*0.34*	*0.72*
	a-	1 (0;2)	1 (0;2)	*1.00*	1 (0;3)	*0.79*	*0.73*	1 (0;2)	*1.00*	*1.00*	*0.69*	1 (0;1)	*0.29*	*0.26*	*0.24*	*0.28*
- Level 2 (<54 mg/dl)	s-	0 (0;0)	0 (0;1)	*0.32*	0 (0;0)	*0.32*	*1.00*	0 (0;0)	*0.32*	*1.00*	*1.00*	0 (0;1)	*0.08*	*0.57*	*0.66*	*0.66*
	a-	0 (0;1)	0 (0;1)	*0.66*	0 (0;1)	*1.00*	*0.66*	0 (0;1)	*0.71*	*1.00*	*0.71*	0 (0;0)	*0.26*	*0.49*	*0.32*	*0.42*
Adherence to HCL use																
Auto mode / Manual mode (%)	s-	- 100	88 (80;92) 13 (8;21)*	***-***	92 (80;97) 7 (3;14)#	***-***	*0.89*	92 (83;96) 9 (4;17)@	***-***	*0.96*	*0.95*	90 (80;95) 10 (5;20)§	*-*	*0.92*	*0.87*	*0.91*
	a-	**-** 100	96 (94;98) 5 (2;6)*	***-***	100 (95;100) 0 (0;5)#	***-***	***0.04***	99 (90;100) 2 (0;10)@	***-***	*0.17*	*0.06*	96 (89;100) 4 (0;11)§	*-*	*0.90*	*0.06*	*0.22*
Sensor wear (%)	s-	90 (84;95)	94 (87;97)	*0.05*	95 (88;97)	*0.12*	*0.79*	93 (89;95)	*0.97*	*0.23*	*0.31*	90 (87;95)	*0.24*	*0.12*	*0.31*	*0.44*
	a-	92 (85;96)	92 (87;97)	*0.24*	92 (87;97)	*0.25*	*0.92*	93 (85;97)	*0.34*	*0.56*	*0.58*	92 (83;95)	*0.88*	*0.20*	*0.13*	*0.21*
Calibrations per day	s-	3.4 (2.8;4.1)	3.4 (3.1;3.9)	*0.55*	3.5 (3.0;4.0)°	*0.48*	*0.74*	3.3 (2.9;3.8)	*0.61*	*0.19*	*0.11*	3.2 (2.4;3.7)	*0.22*	***<0.01***	***0.01***	*0.08*
	a-	4.0 (2.9;5.0)	2.9 (2.7;3.8)	***<0.01***	2.8 (2.4;3.5)°	***<0.01***	*0.24*	2.9 (2.4;3.5)	***<0.01***	*0.30*	*0.74*	2.9 (2.3;3.3)	***<0.01***	***0.01***	*0.20*	*0.14*
Set change every n day	s-	2.5 (2.3;3.2)	2.6 (2.3;2.7)	*0.41*	2.6 (2.2;3.0)	*0.36*	*1.00*	3.7 (2.6;4.5)	***<0.01***	***<0.01***	***<0.01***	3.3 (2.9;3.9)	***<0.01***	***<0.01***	***<0.01***	*0.23*
	a-	2.5 (2.1;3.0)	2.4 (2.0;3.6)	*0.78*	2.6 (2.0;4.0)	*0.36*	*0.51*	3.2 (2.7;4.0)	***0.01***	*0.11*	*0.22*	3.0 (2.5;3.3)	*0.05*	*0.25*	*0.54*	*0.32*
Reservoir change every n day	s-	2.5 (2.3;3.5)	3.0 (2.6;3.0)	*0.29*	3.5 (2.5;3.8)	***0.04***	*0.25*	3.3 (2.7;4.5)	***0.01***	***0.01***	*0.27*	3.3 (2.9;4.0)	***0.01***	***0.01***	*0.57*	*0.39*
	a-	2.5 (2.1;3.0)	2.9 (2.5;4.4)	*0.79*	3.3 (2.5;4.5)	*0.54*	*0.30*	3.2 (2.7;3.7)	*0.94*	*0.63*	*0.18*	3.0 (2.4;3.5)	*0.98*	*0.47*	*0.15*	*0.86*
Insulin dose and basal/bolus ratio																
Total daily dose (U/kg/day)	s-	0.79 (0.61;0.97)	1.04 (0.75;1.14)	***<0.01***	0.85 (0.71;1.06)	***0.04***	***0.04***	0.94 (0.76;1.09)	***<0.01***	*0.32*	*0.29*	0.90 (0.67;1.06)	*0.08*	*0.08*	*0.92*	*0.29*
	a-	0.74 (0.56;0.84)	0.83 (0.70;1.03)	***<0.01***	0.88 (0.73;0.96)	***<0.01***	*0.89*	0.78 (0.70;0.97)	***0.01***	*0.37*	*0.20*	0.82 (0.73;0.96)	***<0.01***	*0.99*	*0.88*	*0.23*
Basal amount / Bolus amount (%)	s-	47 (40;57) 53 (43;61)	46 (43;59) 54 (41;57)†	*0.70*	54 (44;66) 47 (34;57)‡	*0.10*	*0.12*	53 (38;59) 47 (41;62)^	*0.40*	*0.59*	***0.17***	50 (41;60) 50 (40;59)∨	*0.58*	*0.71*	*0.08*	*0.76*
	a-	43 (38;54) 57 (47;62)	42 (38;48) 59 (52;62)†	*0.65*	41 (31;47) 58 (52;62)‡	*0.07*	*0.08*	42 (36;46) 58 (54;64)^	*0.35*	*0.44*	*0.14*	42 (35;48) 58 (52;65) ∨	*0.48*	*0.60*	***0.04***	*0.61*
Auto-correction (%)	a-	–	26 (21;37)	*-*	38 (28;50)	*-*	***0.01***	33 (22;39)	*-*	*0.69*	***0.04***	33 (21;39)	*-*	*1.00*	***<0.01***	*0.55*
Meals and carbohydrates (CHO) intake																
Meals per day	s-	3.8 (2.9;4.4)	4.9 (3.8;6.6)¶	***<0.01***	4.8 (3.3;6.5)	***<0.01***	*0.72*	4.9 (3.2;5.8)	***0.02***	*0.35*	*0.47*	4.9 (4.0;6.9)	***<0.01***	*0.17*	*0.14*	*0.12*
	a-	3.8 (3.0;4.7)	4.2 (3.7;4.9)¶	*0.49*	4.3 (3.2;5.1)	*0.10*	*0.22*	4.4 (3.6;5.2)	*0.13*	*0.28*	*0.83*	4.8 (3.9;5.5)	***0.01***	***0.04***	*0.25*	*0.08*
CHO entered per day (grams)	s-	168 (104;206)	180 (148;264)	***0.01***	181 (139;235)	***<0.01***	*0.32*	163 (109;212)	*0.23*	*0.24*	*0.43*	166 (141;250)	*0.06*	*0.59*	*0.82*	*0.34*
	a-	164 (106;217)	182 (114;215)	*0.92*	167 (121;245)	*0.73*	*0.40*	178 (104;203)	*0.72*	*0.74*	*0.20*	183 (124;224)	*0.87*	*0.73*	*0.76*	*0.38*
CHO intake (grams/kg/day)	s-	3.3 (2.5;4.8)	4.1 (2.9;5.7)	***0.03***	3.8 (2.5;5.0)	*0.06*	*0.15*	3.5 (1.9;5.2)	*0.58*	*0.13*	*0.43*	3.4 (2.2;5.6)	*0.40*	*0.21*	*0.80*	*0.56*
	a-	3.6 (2.5;5.6)	3.8 (2.6;5.6)	*0.81*	3.9 (2.4;5.5)	*0.36*	*0.37*	3.9 (2.1;5.3)	*0.16*	*0.09*	*0.44*	3.7 (2.5;5.3)	*0.28*	*0.32*	*0.99*	*0.39*

Bold values mean significant difference. s-HCL vs. a-HCL, *, # and ‡ p<0.01; § p=0.01; ° p=0.02; § and @ p=0.03; † and ¶ p=0.04.

CHO, carbohydrates; GMI, glucose management indicator; TAR, time above range; TBR, time below range; TIR, time in range.

**Figure 2:**
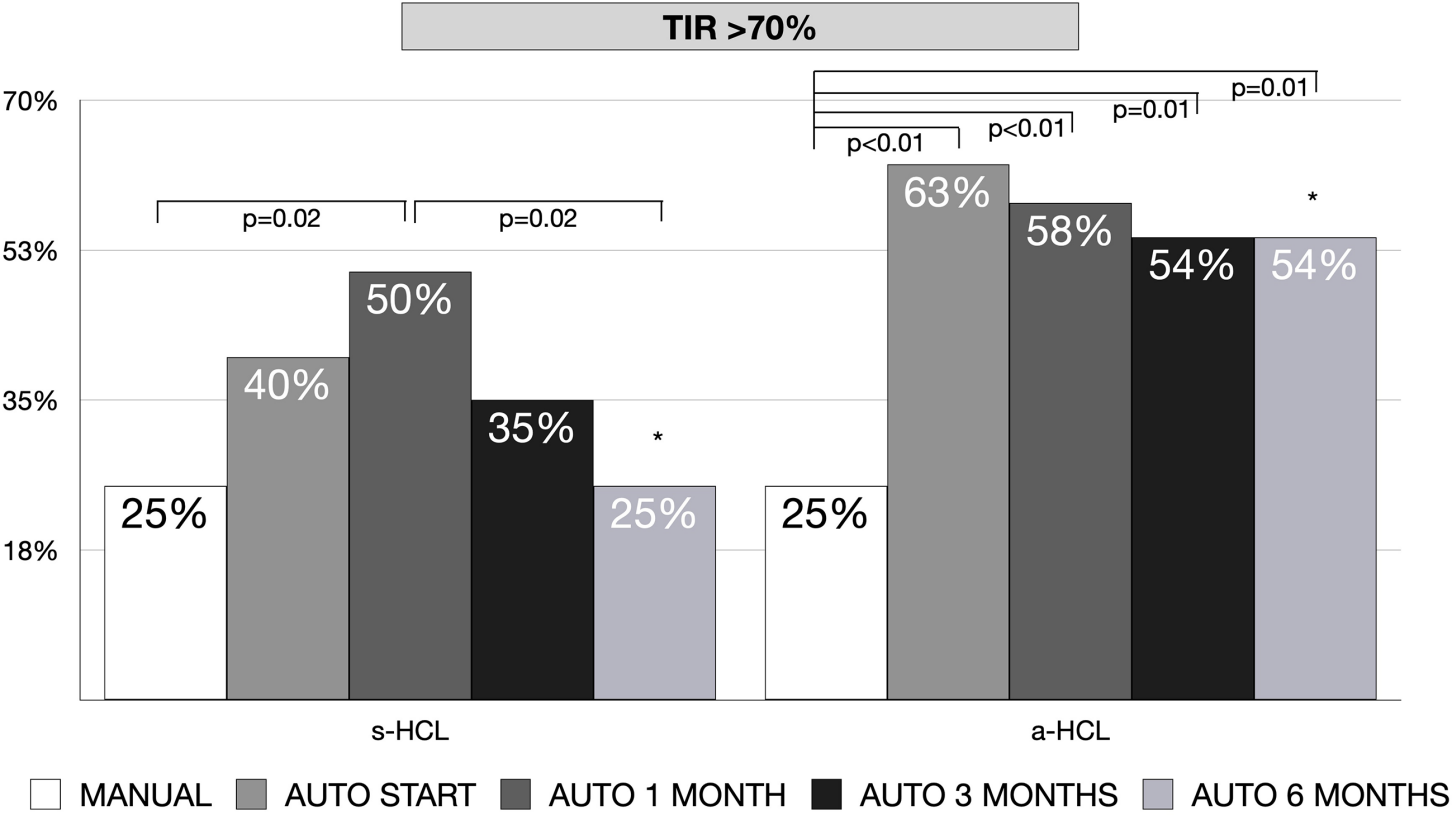
Percentage of patients with TIR (70–180 mg/dL)>70% at the beginning of HCL (AUTO START), after 1 month (AUTO 1 MONTH), after 3 months (AUTO 3 MONTHS) and after 6 months (AUTO 6 MONTHS) of HCL use, compared to baseline in manual mode (MANUAL), divided by type of used hybrid closed-loop (HCL) system [standard (s-HCL) or advanced (a-HCL)]. * AUTO 6 MONTHS s-HCL vs. a-HCL, p=0.04.

The increase in TIR was of a median of 7% (IQR -2;19) for s-HCL and 14% (IQR 7-26) in a-HCL (p=0.20). In a-HCL users, there was a significant increase in the percentage of individuals with TIR>70% from 25% at MANUAL to 63% at AUTO START (p<0.01), remaining stable at subsequent time points. In s-HCL users, the increase was significant from MANUAL (25%) to AUTO 1 MONTH (50%, p=0.02), but not to other time points, with a decline over time, returning to 25% at AUTO 6 MONTHS. The difference between percentage of individuals with TIR>70% at 6 months between a-HCL and s-HCL was statistically significant (54% *vs.* 25%, p=0.04) (**Figure 2**).

For s-HCL users, at linear regression, the rate of bolus amount and the quantity of CHO inserted predicted a TIR>70%; however, at multivariate analysis, only a higher rate of bolus (p=0.04) predicted a TIR >70%. For a-HCL users, at linear regression, the inserted glycemic target, age, total daily insulin, bolus rate, auto-correction rate, number of meals inserted, and quantity of CHO inserted were associated with a TIR>70%; however, at multivariate analysis, only younger age (p<0.01), a higher rate of auto-correction (p=0.03) and a lower amount of CHO inserted (p=0.02) predicted a TIR>70%.

### Changes in Adherence to HCL Use, Insulin Dose, and Basal/Bolus Ratio

A significant difference in time spent in Auto mode was found between a-HCL and s-HCL users at all time points. The decrease in the number of calibrations per day over the study period was significant for a-HCL users only. The interval between set changes and reservoir changes significantly increased throughout the study period for both s-HCL and a-HCL users. No significant changes in sensor wear were found. (**Table 3**).

Total insulin daily dose significantly increased from MANUAL to AUTO START for both s-HCL and a-HCL users. The rate of the basal amount was significantly higher in s-HCL users from AUTO START to AUTO 6 MONTHS. The rate of auto-correction for a-HCL significantly increased from AUTO START to AUTO 1 MONTH (26 to 38%, p=0.01) and then significantly decreased at AUTO 3 MONTHS on (33%, p=0.04).

### Changes in Meals, Carbohydrates (CHO) Intake, and BMI

Meals and CHO entered per day, and CHO intake significantly increased from Manual mode to Auto mode use in s-HCL users only. The number of meals per day was significantly lower in a-HCL than s-HCL (4.2 *vs.* 4.9) at AUTO START only (**Table 3**).

BMI SDS did not significantly change throughout the study period: from a median of 0.57 (IQR -0.35;1.48) at BASELINE to a median of -0.05 (IQR -0.50;1.07) at 3 MONTHS to a median of -0.04 (IQR -0.42;1.18) at 6 MONTHS for s-HCL users; from a median of 0.38 (IQR -0.23;1.60) at BASELINE to a median of 0.40 (IQR 0.09;1.44) at 3 MONTHS to a median of 0.28 (IQR -0.17;1.46) at 6 MONTHS for a-HCL users. The median change in BMI SDS between the last follow-up visit and pre-training visit was not associated with the number of meals or CHO intake.

## Discussion

In this retrospective study, we analyzed data from 44 pediatric subjects with T1DM using HCL systems, showing for the first time in a real-life study that the use of a-HCL for six months significantly reduced HbA1c compared to s-HCL. Users of a-HCL had a lower HbA1c (7.1%) at the 6-month follow-up visit and a more significant decline during its use (-0.5%) compared to s-HCL users (7.7% and -0.1%, respectively). This improvement in HbA1c expands in real-world the findings of the FLAIR crossover (670G/780G) trial (conducted in 113 individuals between 14 and 29 years of age) that reported a significant reduction in HbA1c (-0.5%) between baseline and three months of a-HCL use compared to s-HCL (-0.3%) (20).

The significant differences detected in HbA1c for a-HCL users were not found in sensor-specific measures of glycemic control (such as GMI, TIR, and TAR), possibly due to the different time frame of evaluation (three months in HbA1c *vs.* two weeks in downloaded data). The increase in TIR from Manual mode to 6 months of HCL use was 14% in a-HCL compared to 7% in s-HCL, although this difference was not statistically significant. Nevertheless, the achieved TIR at six months (72%) was better than what reported in the 6-month clinical trials of 670G in children 7-13 years (from 56 to 65%) (26) and adolescents 14-21 years (from 60 to 67%) (7), and in FLAIR trial in s-HCL (from 57 to 63%) and a-HCL users (from 57 to 67%) (20). When considering recommended target (TIR >70%) (27), the users of a-HCL attained this target earlier (63% at the beginning of a-HCL use) and more stably (54% after six months), compared to s-HCL (40% at the beginning and 25% after six months). This can be seen as “the HCL virtuous circle”: when HCL is used correctly, a prompt return to efforts can be perceived with a better quality of life and a reduction in the mental burden of diabetes (23). Moreover, in our cohort, a younger age, a higher rate of auto-correction, and a lower amount of CHO inserted significantly predicted a TIR>70% in a-HCL users.

Since the 780G launch in October 2020, only one real-life 1-month study has been published by Beato-Víbora et al. on 52 T1DM adolescents and adults, showing a rapid improvement in TIR (+12%) without changes in TBR (21). However, the study did not compare with s-HCL systems; moreover, it was based only on downloaded data with a one-month follow-up, and was conducted in well-controlled, experienced SAP-PLGS users aged >15 years.

Another study by Messer et al. on downloaded data in a real-world pediatric population (median age 14 years) treated with an a-HCL based on a different algorithm (Tandem t:slim X2 insulin pump with Control-IQ technology) found results somewhat similar to ours: an increase in TIR from 57% at baseline to 66% at six months (+9%), doubling the proportion of subjects reaching TIR >70% (from 24 to 48%) and improving GMI from 7.5% at baseline to 7.1% at three months and 7.2% at six months (28).

On a glycemic outcome such as HbA1c, our data showed that children between 7 and 14 years of age (not included in the FLAIR study) would benefit the most from a-HCL pumps. Interestingly, a reduction of HbA1c (-0.3%) at six months was found in children aged <7 years, off-label users of a-HCL, although numbers were too small (n=4) to detect any statistical significance. These findings extend what was reported by Salehi et al. in 16 children aged <7 years using s-HCL (reduction of -0.5% in HbA1c over three months) (29).

Remarkably, in our cohort, individuals with a worse glycemic control (>8%) benefited substantially from a-HCL use, from 8.5% at baseline to 7.1% after six months, also when compared to s-HCL (from 8.3 to 8.1%). After three months, no significant differences were found in HbA1c between this group and those with suboptimal (HbA1c 7-8%) and optimal (<7%) control. Previously, only Berget et al. reported that 27 young people with HbA1c >9% using s-HCL declined from 10.7% at baseline, 9.7% at three months, and 9.3% at six months (14). On the other hand, individuals with an HbA1c <7% at baseline using s-HCL tended to worsen glycemic control (6.7 to 7.3%). Interestingly, previous users of s-HCL systems did not show any further improvement with the use of a-HCL. Apart from higher HbA1c at baseline, however, only using an a-HCL system predicted a more significant reduction in HbA1c.

An additional interesting finding is that the use of any HCL system did not lead to an increase in BMI SDS (20). Contrarily to what was reported by Messer et al. (28), in our cohort, the numbers of user-initiated meal insulin boluses significantly increased after using Auto mode, while CHO intake remained stable: children and adolescents using these HCL systems were more prone to bolus for every meal or snack, without increasing the CHO intake, and this an essential indicator of self-management behavior, strongly associated with glycemic control (30, 31). Moreover, no patients discontinued system use during the study period [as reported by Varimo et al. (15)], compared to 4-30% of discontinuations reported in other cohorts (14, 28). This can be explained by the high motivation of children, adolescents, and their families who started the HCL training in our Institute and their commitments by signing a therapeutic contract at the beginning (23). This is further confirmed by the excellent adherence to HCL use in these subjects (14): the sensor wear and the use of Auto Mode did not decline significantly over the study period and were >90% after six months.

The main limitation of our study is the retrospective design. Although randomized controlled trials are the gold standard for evaluating treatment outcomes, providing information on treatments’ “efficacy”, their strict and controlled conditions lead to low generalizability because they might be very different from usual real-life care. Conversely, real-life studies inform on the “effectiveness” of treatment, the measure of the extent to which that intervention does what is intended to do in ordinary circumstances (32). Our data support the idea that a-HCL may perform better than s-HCL as well in clinical practice as it did in clinical trials.

Moreover, the comparison of sensor-specific measures of glycemic control was made towards Manual mode. Manual mode is challenging with significant adjustments required; therefore, a comparison with data from before any type of HCL/manual mode would have been more indicative of the difference between being on an HCL system and not being on an HCL system. Another limitation is that these data reflect an experience in a high-standard health care system. Thus, these results may not wholly be generalized to other health care systems or every child or adolescent with T1DM. However, this limitation can be restrained when using HCL systems since the algorithm makes a large part of the work.

The study’s strengths are the real-life conditions and the heterogeneous population, with no strict exclusion criteria on HbA1c levels or treatment modalities before HCL use that reflect everyday practice. Moreover, to the best of our knowledge, this is the first post commercial real-world analysis of the effectiveness of the a-HCL Minimed 780G system in children <14 years with T1DM, providing initial data on a very complex group such as pre-school children. Although it should be confirmed in larger cohorts, the proof of safety and effectiveness of the system in this subgroup may lead to removing the current age limitation, leaving only the minimum daily dose ≥ of 8 units required by the algorithm.

## Conclusions

This real-world study suggests that effectiveness might be greater in a-HCL than in s-HCL, with significant changes in HbA1c and reaching earlier and more stably the target of TIR >70%, without increasing hypoglycemia or BMI. At the same time, previous users of s-HCL systems did not show any further improvement with a-HCL. Children under the age of 14 years of age, not represented in previous studies, seem to benefit the most from a-HCL pumps as well as individuals with the worst glycemic control.

## Data Availability Statement

The raw data supporting the conclusions of this article will be made available by the authors, without undue reservation.

## Ethics Statement

Ethical review and approval was not required for the study on human participants in accordance with the local legislation and institutional requirements. Written informed consent to participate in this study was provided by the participants’ legal guardian/next of kin.

## Author Contributions

GT conceptualized and designed the study, drafted the initial manuscript, designed the data collection instruments, carried out the statistical analysis and reviewed and revised the manuscript. FB drafted the initial manuscript, designed the data collection instruments, collected data and reviewed and revised the manuscript. CC and EF were involved in the clinical care of the individuals and contributed in writing and revising the manuscript. EB critically reviewed the manuscript for important intellectual content. All authors approved the final manuscript as submitted and agree to be accountable for all aspects of the work.

## Conflict of Interest

The authors declare that the research was conducted in the absence of any commercial or financial relationships that could be construed as a potential conflict of interest.

## Publisher’s Note

All claims expressed in this article are solely those of the authors and do not necessarily represent those of their affiliated organizations, or those of the publisher, the editors and the reviewers. Any product that may be evaluated in this article, or claim that may be made by its manufacturer, is not guaranteed or endorsed by the publisher.
